# Small-scale spatial variation in population- and individual-level reproductive parameters of the blue-legged hermit crab *Clibanarius tricolor*

**DOI:** 10.7717/peerj.3004

**Published:** 2017-02-15

**Authors:** J. Antonio Baeza, Donald C. Behringer

**Affiliations:** 1 Department of Biological Sciences, Clemson University, Clemson, SC, United States; 2 Smithsonian Marine Station at Fort Pierce, Fort Pierce, FL, United States; 3 Departamento de Biologia Marina, Universidad Catolica del Norte, Coquimbo, IV Region, Chile; 4 School of Forest Resources and Conservation, University of Florida, Gainesville, FL, United States; 5 Emerging Pathogens Institute, University of Florida, Gainesville, FL, United States

**Keywords:** Egg production, Fecundity, Abundance, Crustacea

## Abstract

Management of the few regulated ornamental fisheries relies on inadequate information about the life history of the target species. Herein, we investigated the reproductive biology of the most heavily traded marine invertebrate in the western Atlantic; the blue-legged hermit crab *Clibanarius tricolor*. We report on density, individual-level, and population-level reproductive parameters in 14 populations spanning the Florida Keys. In C. tricolor, abundance, population-level, and individual-level reproductive parameters exhibited substantial small-scale spatial variation in the Florida Keys. For instance, the proportion of brooding females varied between 10–94% across localities. In females, average (±SD) fecundity varied between 184 (±54) and 614 (±301) embryos crab-1 across populations. Fecundity usually increases with female body size in hermit crabs. However, we found no effect of female body size on fecundity in three of the populations. Altogether, our observations suggest that C. tricolor may fit a source-sink metapopulation dynamic in the Florida Keys with low reproductive intensity and absence of a parental body size—fecundity relationship resulting in net reproductive loses at some localities. We argue in favor of additional studies describing population dynamics and other aspects of the natural history of C. tricolor (e.g., development type, larval duration) to reveal ‘source’ populations, capable of exporting larvae to nearby populations. Our observations imply that future studies aimed at assessing standing stocks or describing other aspects of the life history of this hermit crab need to focus on multiple localities simultaneously. This and future studies on the reproductive biology of this species will form the baseline for models aimed at assessing the stock condition and sustainability of this heavily harvested crustacean.

## Introduction

Ornamental organisms support a multi-million dollar global industry that provides marine and freshwater aquarist with an assortment of over 1,400 species of invertebrates, vertebrates, plants, and algae (Chapman et al., 1997; Calado et al., 2003; Rhyne et al., 2009). Many ornamental fisheries operate unnoticed due to their niche market, the frequency of product export, and the small size attained by the target organisms. Although numerous, ornamental fisheries have historically received minor attention from a resource-management standpoint. Furthermore, management of the few regulated ornamental fisheries relies on limited information about the life history of the target species (Calado, 2008; Rhyne et al., 2009). The marine industry most often depends upon harvesting of organisms from the natural environment. The extraction of ornamentals has intensified remarkably during the last two decades (Green, 2003; Calado et al., 2003; Rhyne et al., 2009), and this fishing effort likely will continue to increase during the coming years (Wood, 2001). It is therefore urgent to develop a baseline of biological information so marine ornamental fisheries can be managed sustainability.

In North America, the state of Florida leads the extraction and export of marine ornamentals (Rhyne et al., 2009). Over 600 fish, invertebrates, and plant species are extracted every year from intertidal and shallow coastal waters, in particular from the Florida Keys, and approximately 35% of those are decapod crustaceans (Rhyne et al., 2009). Examples include boxer shrimps from the genus *Stenopus*, true crabs from the species complex *Mithrax*—*Mithraculus*, caridean shrimps from the genera *Lysmata, Ancylomenes*, and *Periclimenes*, and hermit crabs from the genera, *Dardanus*, *Petrochirus*, and *Clibanarius* (Calado et al., 2003; Calado, 2008; Rhyne et al., 2009; Baeza et al., 2012; Baeza et al., 2013). The aim of this study was to investigate the reproductive biology of the most heavily traded marine invertebrate in the western Atlantic, *Clibanarius tricolor* (Gibbes, 1850).

**Figure 1:**
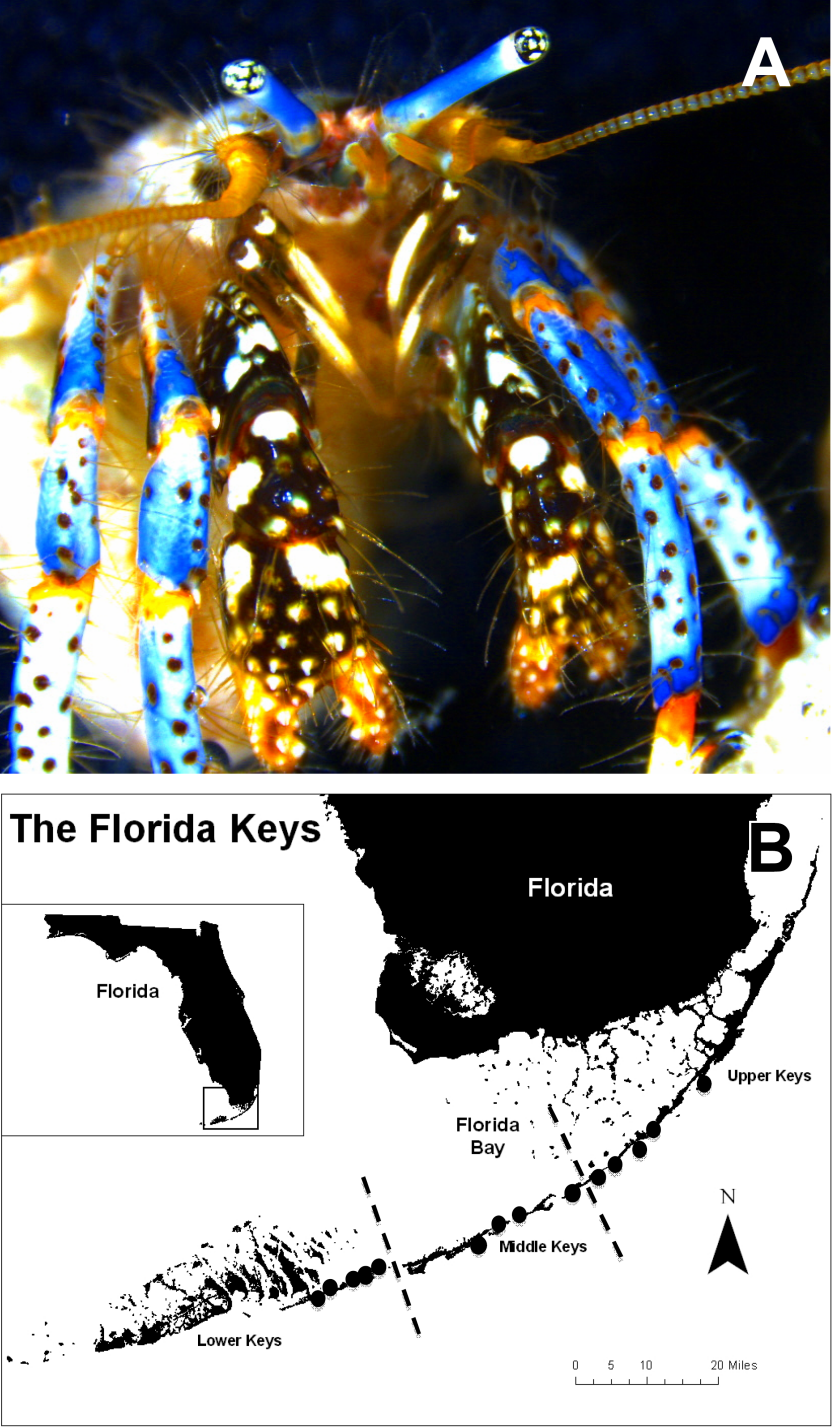
*Clibanarius tricolor* (Gibbes, 1850). (A) hermit crab anterior view; (B) Map of the Florida Keys, USA, showing the survey/sampling sites for the blue-legged hermit crab *Clibanarius tricolor*. Black dots indicate the general area of study. From northeast to southwest, the study sites were Harry Harris, Islander Resort, Indian Key Fill, Lignumvitae Channel, Anne’s Beach, Mile Marker 75, Tom’s Harbor, Long Key Bank, Rainbow Bend Resort, Money Key, Missouri Key, Ohio-Missouri Key, Mile Marker 35, and Scout Key.

In 2007, marine life licensed fishers harvested 2.4 million *C. tricolor* individuals (FWC, 2007 landing data). Among hermit crabs, the blue-legged hermit crab *C. tricolor* is highly valued both because of its aesthetic value and apparent ability to control the small sea anemone *Aiptasia* spp., considered a nuisance by aquarium hobbyists (Calado, 2008; Rhyne et al., 2009). *Clibanarius tricolor* is a small hermit crab commonly found in the intertidal zone and shallow subtidal (up to ∼2 m) throughout the Caribbean Sea and western Atlantic (Provenzano, 1960) (Fig. 1). It can form temporarily large aggregations during low tides in the intertidal zone where it finds refuge in depressions beneath rocks on hard-bottoms. It can also be found in intertidal sandy-bottom substrates (Provenzano, 1960) and shallow subtidal seagrass meadows dominated by *Thalassia testidinium* (Bauer, 1985). It uses shells from various gastropods but most often *Cerithium* spp. and appears to be more active at night than during day hours (Hazlett, 1983; Bauer, 1985). Field experiments have shown that *C. tricolor* exhibit high site fidelity and do not make large daily movements, having an average home range no larger than 2 m in diameter (Hazlett, 1983). Lastly, *C. tricolor* overlaps strongly in shell utilization with other sympatric hermit crabs in the Florida Keys and field observations indicate that one sympatric species, *Calcinus tibicen* (Herbst, 1791), dominate *C. tricolor* in shell fights impacting its shell use and fecundity (Bach, Hazlett & Rittschof, 1976).

Although, our knowledge on the biology of *C. tricolor* has improved during recent decades, many reproductive, behavioral, physiological, ecological, and natural history aspects of this species are still unknown. For instance, very little is known about population- and individual-level reproductive parameters and small- and large-scale spatial variability of these parameters, although this kind of information is vital to manage any fishery sustainably (King & McFarlane, 2003; (Winemiller, 2005)). Thus, we set out to examine the abundance, population-level (i.e., size-frequency distribution, sex ratio), and individual-level (i.e., fecundity, egg size, reproductive output) reproductive parameters of this species at small spatial scales (<1 km^2^) at 14 sites distributed along the Florida Keys, USA. We focused on small spatial scale variability in reproductive parameters given the small home range, small body size, and high habitat fidelity of the species—traits that make *C. tricolor* prone to small-scale spatial variation in reproductive traits. This and future studies on the reproductive biology of this species will form the baseline for models aimed at assessing the stock condition and sustainability of this heavily harvested ornamental crustacean.

## Material and Methods

### Sampling sites and sampling of *Clibanarius tricolor*

To investigate small-scale spatial variation in hermit crab population structure and reproductive traits, we subdivided the Florida Keys into 3 geographic strata: Lower Keys (Key West to Seven-mile Bridge), Middle Keys (Seven-Mile Bridge to Lower Matecumbe), and Upper Keys (Lower Matecumbe through Key Largo). Within each larger stratum we selected 4–5 survey sites (Fig. 1). Sites were selected based on knowledge of habitat preference for the species, preliminary observations, and NOAA benthic habitat maps.

### Population density and distribution of *Clibanarius tricolor*

Field collections were approved by FWCC (permit number: 06021510). At each site we haphazardly placed 15 PVC pipe quadrats (0.5 m^2^) within shallow hard-bottom habitat. We also tested smaller 0.25 m^2^ quadrats simultaneously, but found them to be of inadequate size (too many were devoid of crabs). From each 0.5 m^2^ quadrat we collected all of the gastropod shells, regardless of their contents. In addition to describing the population structure of blue-legged hermit crab, this enabled us to calculate the availability, size, and types of shell used by *C. tricolor*. Although quadrats were placed haphazardly, we attempted to characterize the entire area at each survey site.

To evaluate differences in habitat use, we also measured the benthic coverage at each site using a line-intercept method. At each site we haphazardly laid out four different 10 m measuring tapes along the substrate. We then moved along each tape and recorded the bottom type over which the tape was laid. We only considered bottom type patches >10 cm. The benthos was characterized as sand, gravel, rubble (rocks > 5 cm), boulder (rocks > 10 cm), bedrock, algae, or seagrass. We then calculated the proportion of each bottom type and analyzed the relationships between the bottom types and the total crab abundance site^−1^ and mean density quadrat^−1^ site^−1^ using a stepwise regression analysis. The best fit model was selected using the minimum corrected Akaike Information Criterion (AICc).

To examine the population distribution of *C. tricolor*, we used two different indices of dispersion suitable for frequency distributions calculated from quadrat counts, the variance/mean ratio and Morisita’s Standardized Index. For the variance/mean ratio a value of 0 indicates a uniform distribution, 1 indicates random distribution, and >1 indicates a clumped distribution. For Morisita’s Standardized Index, the range is from −1 to +1, with calculates >0 indicating clumping, 0 indicating uniformity, and 0 indicating a random distribution (Elliott, 1983).

Diversity and availability of gastropod shells are known to affect population parameters in other species of hermit crab (Fotheringham, 1976; Elwood, Marks & Dick, 1995; Iossi, Garcia & Mantelatto, 2005, and references therein). In the case of *C*. *tricolor*, preliminary observations across populations in the Florida Keys indicated that *Cerithium* spp. was either the single or most predominantly used shell type in the region. Given this predominant use of *Cerithium* spp. by *C. tricolor*, we decided not to estimate the effect of shell use on population and reproductive parameters in the studied populations. Also, logistic constraints did not permit us to estimate shell abundance and type in the different studied populations.

### Population- and individual-level reproductive traits in *Clibanariustricolor*

For the analysis of population- and individual-level reproductive parameters, between 100–150 individuals of *C*. *tricolor* were haphazardly collected from 9 of the 14 different sites in the Florida Keys between July 2011 and January 2012. Due to logistical constraints, we choose only three localities each in the Upper (i.e., Harry Harris, Indian Key Fill, and Islander Resort), Middle (Long Key Bank, Mile Marker 75, and Tom’s Harbor Key) and Lower Keys (Money Key, Ohio-Missouri Channel, and Scout Key) (Fig. 1).

Immediately after collection, crabs were transported alive to the Goshen Marine Laboratory located on Long Key, FL, and fixed in 10% neutral buffered formalin. Next, all gastropod shells used as refuge by hermit crabs were identified to the species level and measured (maximum opercula length, mm) with a manual dial caliper (to the nearest 0.1 mm) before gently cracking them open using a commercial vise. We were careful to avoid damaging the crabs during the removal procedure. Next, each hermit crab was sexed and measured under a stereomicroscope. Sex was determined according to the presence/absence of external primary sexual traits (i.e., gonopores located at the base of the coxae of the third pair of pereopods in males and fifth pair of pereopods in females). The shield length (SL) of each hermit crab was measured to the nearest 0.05 mm. Intersex individuals are known to occur with a low frequency in natural populations of *Clibanarius*, including *C. tricolor* including the FL Keys (see Data S1). We did notice these intersex individuals at extremely low frequencies. However, we are not reporting these data here but in a future paper together with additional information on the reproductive biology of these uncommon individuals.

### Population- and individual-level reproductive traits in *Clibanariustricolor*

We determined sex ratio (males/males + females), proportion of brooding females (brooding females/brooding + non brooding females), and size at first maturity of *C*. *tricolor* at the different studied localities. The observed sex ratio was compared and tested for deviations from a 1:1 sex phase ratio using the binomial test (Wilson & Hardy, 2002). We attempted to estimate size at first maturity considering the relative proportion of females with and without embryos across body size classes using logistic regression (Wilson & Hardy, 2002). However, this was precluded by the large number of non-brooding females at most study sites, including those amongst the largest body size classes (see results for details).

Fecundity, reproductive output and embryo size were estimated in all brooding females collected at each sampling site. When possible, female crabs carrying newly spawned (early) embryos were selected to cover most of the range in body size observed for the species. Embryos were gently collected with forceps from pleopods in the abdomen, measured (widest and longest axis each of ten embryos per female) and then counted under a stereomicroscope (Wild Co. Model M5A). Direct counts of the embryos were performed on females carrying 400 or less embryos. In females carrying more than 400 embryos, four sub-samples of 100 embryos were separated from the brood mass. Next, the female crab, sub-samples, and the remaining of the embryo mass were dried for 48 h at 70 °C in a drying oven (LO–201C; Grieve Co.), and weighed to the nearest 0.01 mg with an analytical balance (Mettler AE163). Fecundity in these females was estimated as in Baeza et al. (2016) with the formula: *N* = *EM*∕(*SS*_1_ + *SS*_2_ + *SS*_3_ + *SS*_4_)∕400 + 400. Where *N* = total number of embryos per female, *EM* = dry weight of the remaining embryo mass after the four sub-samples (*SS*_1_–*SS*_4_) have been extracted. Egg volume was estimated with the formula for an ellipsoid *EV* = *πLS*^2^∕6, where *L* = long axis and *S* = short axis (Turner & Lawrence, 1979).

Reproductive output was estimated as the ratio between dry weight of the embryos and dry weight of the female carrying the embryos. This reproductive output represents the amount of resources that females invest into reproduction (Hines, 1982; Baeza et al., 2016). We tested whether or not reproductive output increases linearly with body size of females. The relationship between reproductive output and body dry mass of females was examined using the allometric model *y* = *ax*^*b*^ (Hartnoll, 1982). The slope b of the log–log least-squares linear regression represents the rate of exponential increase (*b* > 1) or decrease (*b* < 1) of the estimate of reproductive allocation with a unit of increase in crab dry mass. To determine if the relationship deviates from linearity, an *F*-test or *t*-test was used to test if the estimated slope b deviates from the expected slope of unity (Zar, 1999). Before conducting the test, assumptions of normality and homogeneity of variances were checked and found to be satisfactory. We determined whether or not EV increased with SL. For this purpose, we determined if the correlation between EV and SL was significantly different from zero using different regression analyses (Zar, 1999). Lastly, we used separate ANCOVA and GLM analyses to compare the effect of locality and female body size on the different female-specific reproductive parameters above across the Florida Keys.

## Results

### Population structure, abundance, and distribution of *Clibanariustricolor*

**Figure 2:**
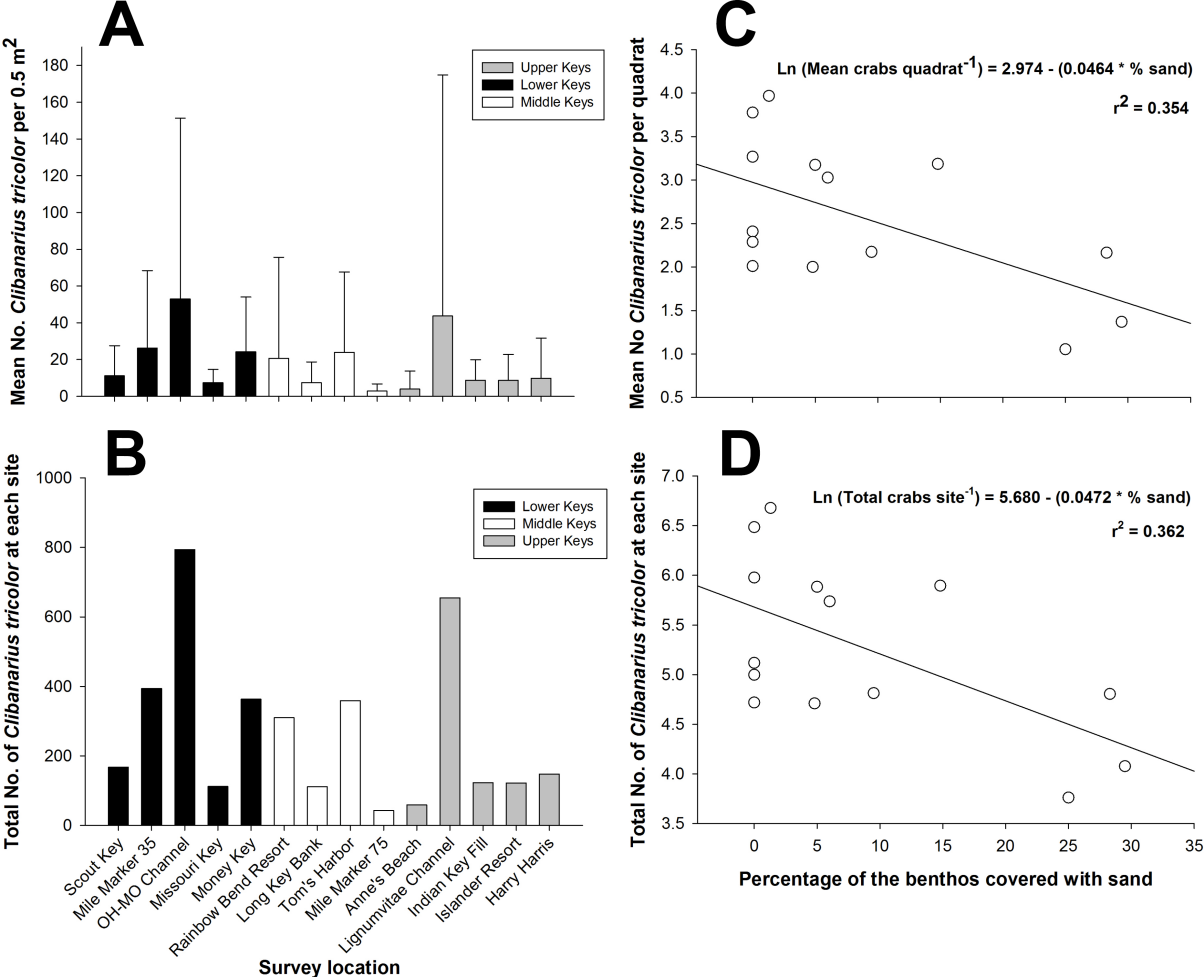
*Clibanarius tricolor* (Gibbes, 1850). (A) Mean number of hermit crabs per 0.5 m^2^ quadrat at each site in the lower, middle, and upper Florida Keys. Bars represent ±1 standard deviation. (B) Total number of *C. tricolor* from 15 quadrats (0.5 m^2^) at each site in the lower, middle, and upper Florida Keys. (C) Plot of the mean abundance of *C. tricolor* versus the percentage of sand covering the benthos at each site. Benthic coverage was estimated from four 10 m point-intercept coverage measurements at each site. (D) Plot of the mean *C. tricolor* density 0.5 m^2−1^ versus the percentage of sand covering the benthos at each site. Benthic coverage was estimated from four 10 m point-intercept coverage measurements at each site.

There was considerable variability in population density among study sites throughout the Florida Keys when considering the density per 0.5 m^2^ quadrat (Fig. 2A) and considering the total abundance at each site for the 15 combined 0.5 m^2^ quadrats (Fig. 2B). The two indices of dispersion, variance/mean ratio and Morisita’s Standardized Index, used to reveal the distribution of *C. tricolor* in the Florida Keys revealed that the different populations were aggregated at the 0.5 m^2^ scale (all variance/mean ratio values >92, all Morisita’s Standardized Index values >1.0, *P* > 0.05 in all cases). However, we found no difference in the mean hermit crab abundance between the Lower Keys (*x* = 24.4), Middle Keys (*x* = 11.76), and Upper Keys (*x* = 18.07) (one-way ANOVA: *F* = 1.1379, *df* = 2, 207, *P* = 0.3225), suggesting that although spatially aggregated on a small scale, their population abundance is homogeneous on a broad scale.

**Figure 3:**
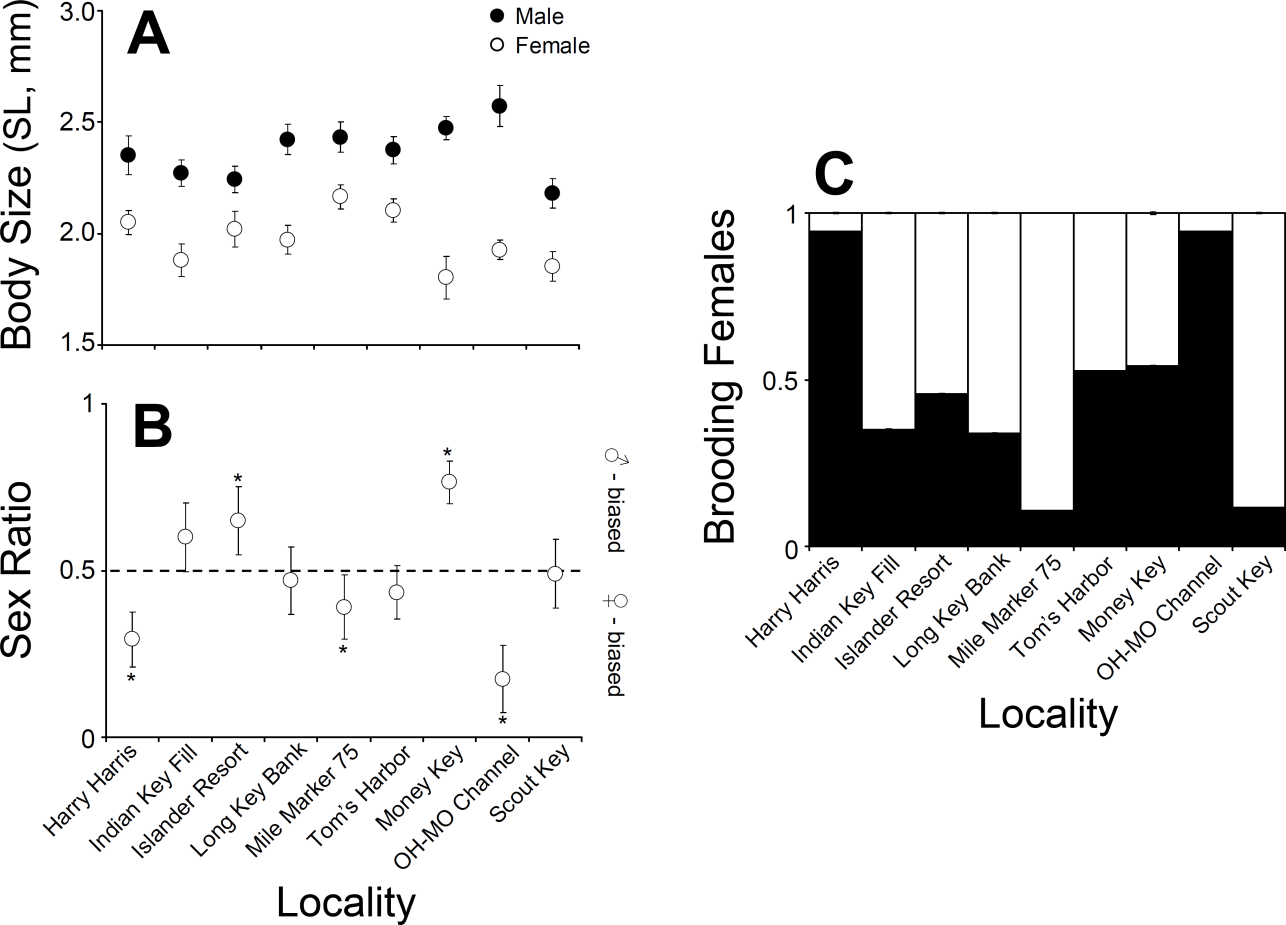
*Clibanarius tricolor* (Gibbes, 1850). (A) Average body size (shield length, mm) of males and females at nine different sampling sites in the upper, middle, and lower Florida Keys. (B) Sex ratio at nine different sampling sites in the upper, middle, and lower Florida Keys. (C) Proportion of ovigerous females at nine different sampling sites in the upper, middle, and lower Florida Keys.

When we examined the relationship between the percentage of the various benthic coverage types and total hermit crab abundance site^−1^ and the mean hermit crab density per quadrat (0.5 m^2−1^), for both total hermit crab abundance (*R*^2^ = 0.3624; AICc =36.8053) (Fig. 2C) and density per quadrat (*R*^2^ = 0.3535; AICc =36.8268) (Fig. 2D) only the percentage of sand covering the benthos was a significant (negative) predictor of total hermit crab abundance. For both analyses the data were natural log transformed to achieve normality.

### Population-level reproductive parameters

Male *C. tricolor* average body size varied between 2.6 (±0.09) mm SL at Ohio-Missouri Channel and 2.2 (±0.07) mm SL at Scout Key. In turn, female average body size varied between 1.8 (±0.09) mm CL at Money Key and 2.17 (±0.05) mm CL at Mile Marker 75, Middle Keys (Fig. 3). A two-way ANOVA using sex and locality as independent variables demonstrated a significant effect of locality and sex in hermit crab body size. Overall, males were larger than females (ANOVA, Sex effect: *F* = 151.2, *df* = 2, 996, *P* < 0.001). Also, hermit crabs collected from Islander Resort, Money Key, Long Bank Key, and Harry Harris were larger those collected from the remaining of the sampling sites. Lastly, crabs collected from Indian Key Fill, Tom’s Harbor, and Ohio-Missouri Channel were larger than those collected from Scout Key and Mile Marker 75 (Locality effect: *F* = 3.78, *df* = 8, 996, *P* = 0.002, and a posteriori comparisons). In the two-way ANOVA, the interaction term was significant (*F* = 2.68, *df* = 8, 996, *P* = 0.0065). This is important because it shows variable sexual dimorphism with respect to body size in *C. tricolor*. For instance, the differences in body size between male and female hermit crabs were much larger at Money Key and Ohio-Missouri Channel than at Islander Resort, where the differences in body size between the sexes were among the smallest observed across the Florida Keys (Fig. 3).

Sex ratio in *C. tricolor* varied between 0.17 (C.I. =0.11–0.27) at Ohio-Missouri Channel and 0.77 (C.I. =0.68–0.86) at Money Key (Fig. 3). At Indian Key Fill, Long Key Bank, Scout Key, and Tom’s Harbor, the observed sex ratio did not differ significantly from a 1:1 sex ratio (Binomial test, *P* > 0.05 in all cases). In turn, at Islander Resort and Money Key, the population was dominated by males (Islander Resort: *P* = 0.0022; Money Key: *P* < 0.0001) while at Harry Harris, Ohio-Missouri Channel, and Mile Marker 75 the population was dominated by females (Harry Harris: *P* < 0.0001; Ohio-Missouri Channel: *P* < 0.0001; Mile Marker 75: *P* = 0.0168) (Fig. 3).

The male biased sex ratio coupled with the relatively large number of females carrying no embryos at most sites precluded the logistic analysis of size at first maturity, both on a per locality basis and even when all the data were pooled together. In general, the logistic regression did not represent a “good” model for our data due to the relatively small number of small females at each site and the large proportion of females not carrying embryos. The body size of the smallest brooding female varied between 1.4 mm SL at Tom’s Harbor and 1.7 mm SL at Ohio-Missouri Channel, Harry Harris, and Indian Key Fill. This size range represents the our best estimation of size at first maturity in *C. tricolor* in the Florida Keys.

Lastly, the proportion of brooding females in *C. tricolor* varied between 0.1 at Mile Marker 75 Key and 0.94 at Harry Harris and Ohio-Missouri Channel (Fig. 3). Visual examination of the data denoted considerable variability in the percentage of brooding females among populations and no particular trend from upper to lower Keys.

### Individual-level reproductive parameters

Given the large number of non-brooding females at several sampling sites, the analyses of size-specific female reproductive parameters were conducted only using data from five of the nine localities in which at least eight females with early stage eggs were observed.

**Figure 4:**
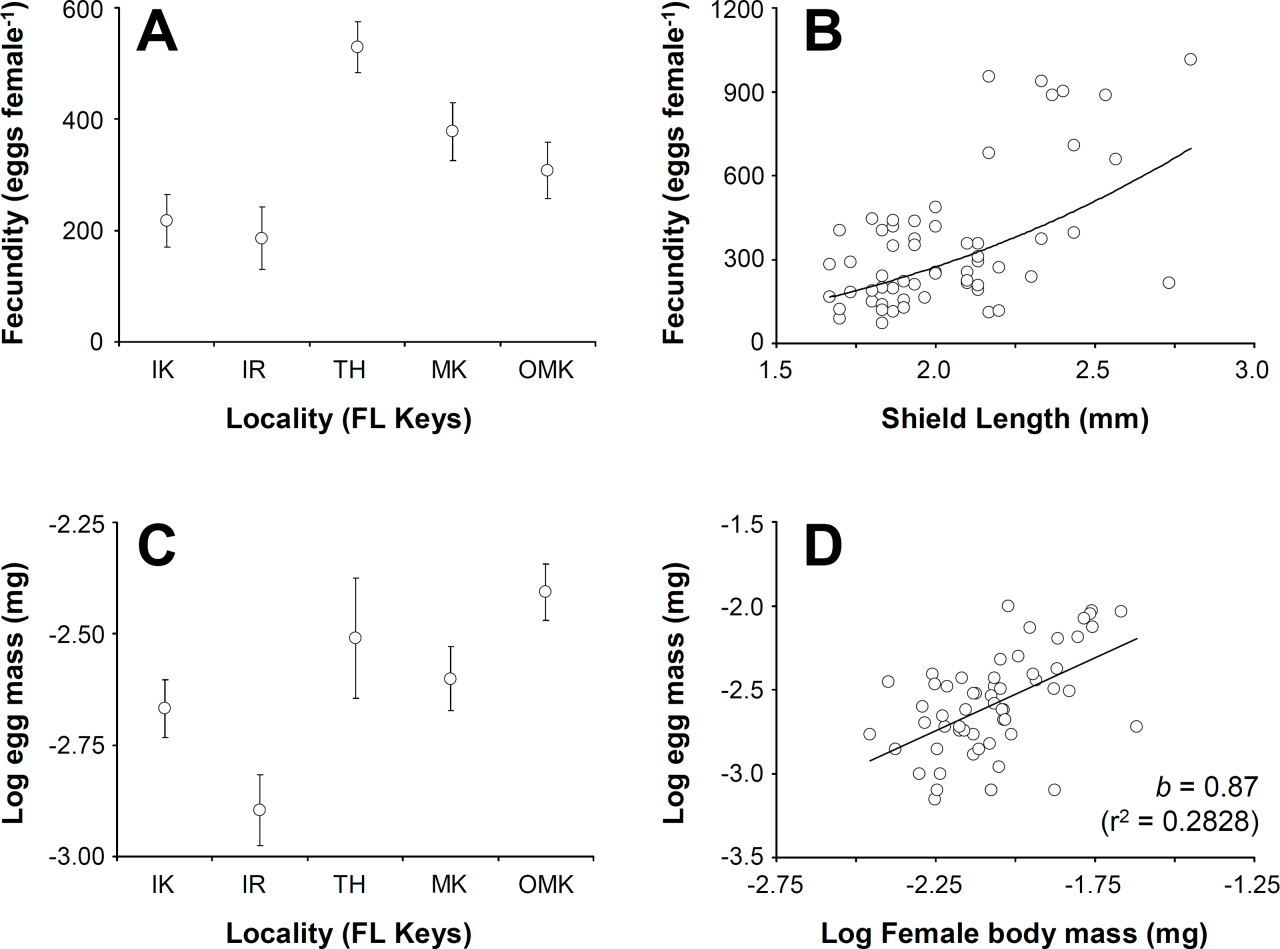
*Clibanarius tricolor* (Gibbes, 1850). Reproductive parameters in brooding females in the Florida Keys. (A) average (±standard error) fecundity in five different populations of *C. tricolor* in the Florida Keys. (B) the relationship between female body size (Shield Length, SL) and fecundity. (C) average (±standard error) reproductive output in five different populations of (C) tricolor in the Florida Keys. (D) the relationship between female dry mass and egg mass dry mass. IK, Indian Key; IR, Islander Resort, TH, Tom’s Harbour; MK, Money Key; and OMK, Ohio-Missouri Key.

Female fecundity (average (±standard deviation, SD)) varied between 184 (±54) embryos crab^−1^ at Indian Key Fill and 614 (±301) embryos crab^−1^ at Tom’s Harbor (Fig. 4A). Unexpectedly, embryo number increased significantly with female SL only at Indian Fill Key and Tom’s Harbor (Indian Fill Key, *t*-test: *t* = 2.22, *df* = 1, 10, *P* = 0.0465; Tom’s Harbor, *t*-test: *t* = 5.29, *df* = 1, 14, *P* = 0.0373). By contrast, no effect of female body size on fecundity was detected at the rest of the study sites. We conducted a one-way GLM using locality as the independent variable, female SL as a covariate and fecundity as the dependent variable to determine the combined effect of locality and body size on fecundity across the Florida Keys. Given that fecundity depended upon body size at some locations but not at others, we predicted a significant effect of the interaction term. However, the GLM produced no significant main effects (Locality: *F* = 0.45, *df* = 4, 48, *P* = 0.7688; female SL: *F* = 1.08, *df* = 1, 48, *P* = 0.3037), nor was the interaction term significant (*F* = 0.72, *df* = 4, 48, *P* = 0.5803). Consequently, we removed the interaction term ran the analysis again. This second GLM demonstrated a significant effect of locality (*F* = 8.44, *df* = 4, 57, *P* < 0.0001) and female body size (*F* = 14.58, *df* = 4, 57, *P* = 0.0004). Overall, fecundity increases with body size in *C. tricolor* and a post-hoc multiple comparisons analysis demonstrated that fecundity at Indian Key Fill and Islander Resort was smaller than that attained by female crabs at Money Key and Ohio-Missouri Key. Also, females attained the largest fecundity at Tom’s Harbor compared to other localities in the Florida Keys (Fig. 4B).

Embryo volume varied between 0.0186 (±0.004) mm^3^ at Money Key and 0.0411 (±0.0114) mm^3^ at Indian Key Fill. No correlation between embryo volume and female body size was found for any of the sites in which the relationship between these variables was studied (regression analysis: *F* < 0.97, *P* > 0.05 in all cases). We conducted a one-way GLM using locality as independent variable, female SL as covariate and egg volume as dependent variable to determine the combined effect of locality and female body size in embryo volume across the Florida Keys. Given that embryo volume did not depend upon female body size at all locations (see above), we predicted no significant effect of the covariate and of the interaction term in the model. In agreement with expectations, the interaction term was not significant (*F* = 0.65, *df* = 4, 48, *P* = 0.6282), so we extracted this term from the model and ran the analysis again. In the second analysis, the GLM detected a significant effect of Locality (*F* = 31.33, *df* = 4, 57, *P* < 0.0001) but, as expected, no significant effect of female body size (*F* = 0.56, *df* = 4, 57, *P* = 0.4547) on embryo volume. A post-hoc multiple comparisons analysis indicated that egg volume was greater at Indian Key Fill compared to the rest of localities sampled.

Lastly, reproductive output represented, on average, between 17.2% (±9.7) and 49% (±24) of female body dry weight at Islander Resort and Tom’s Harbor, respectively (Fig. 4C). At all localities, reproductive output increased linearly with hermit crab body weight as the slope of the line describing the relationship between these two variables was not significantly different from unity (after log–log data transformation, *t*-test: *t* < 0.92, *P* > 0.05 in all cases). An ANCOVA using locality as the independent variable, female dry body weight as the covariate and brood weight as the dependent variable demonstrated an effect of female body size on reproductive output (Female dry weight term: *F* = 7.31, *df* = 1, 47, *P* = 0.0095). Locality did not affect reproductive output (Locality term: *F* = 0.12, *df* = 4, 47, *P* = 0.9728). Also, the interaction term was not significant (*F* = 0.25, *df* = 4, 47, *P* = 0.9081). Thus, reproductive output of *C. tricolor* does not vary across the Florida Keys and females increase allocation to reproduction (in terms of brood mass) proportionally with increasing body size (Fig. 4D).

## Discussion

In the blue-legged hermit crab *C. tricolor*, abundance, population-, and individual-level reproductive parameters exhibited substantial small-scale spatial variation in the Florida Keys. The extent of such variability was unexpected considering the totality of the studied populations did not extend over 100 km of coast.

In *C. tricolor*, the proportion of brooding females in the population varied between <10% to >90% across study sites. At individual-level, egg number increased significantly with female body size in only 2 of 5 populations and fecundity also varied considerably among the studied populations. Furthermore, in all prior studies of which we were aware, fecundity increased with body size in every species of hermit crab (Wait, 2010; Wait & Schoeman, 2012 and references therein). At present, we can only hypothesize regarding the difference we measured in brooding intensity and the lack of an effect of parental body size on fecundity in some but not other populations. Shell type (=species) has been shown to affect egg production in its congener *Clibanarius virescens* (Wait, 2010), but for *C. tricolor* from the Florida Keys, only a single shell type (i.e., *Cerithium* spp.) was predominantly used by crabs. Still, although all individuals were using the same shell type, we can expect competition for the best fitting shells within each population of hermit crab (see Tran et al., 2014). If males, with large body sizes, are dominant over smaller females in shell competition, this could explain both decreased fecundity (and body sizes) in females compared to males in some but not other populations (e.g., with more or less shell availability). Other conditions that could affect the brooding intensity and size-specific fecundity across populations in the Florida Keys include site-dependent food availability, inter-specific competition, predation intensity (via its effect on female foraging behavior), or a combination of the above (Bertness, 1981a; Bertness, 1981b). Certainly, the conditions driving small-scale spatial variation in abundance and reproductive traits herein reported for *C. tricolor* deserve further attention. The remarkable disparity in reproductive performance among populations of *C. tricolor* suggest that this species may actually fit the model of a source–sink metapopulation dynamic (Dias, 1996) in the Florida Keys. The low percentage of ovigerous females and the absence of a body size—fecundity relationship in some of the studied populations might result in *C. tricolor* experiencing net reproductive loses at some localities but not others (Pulliam, 1988; Dias, 1996).

It is worth addressing two issues rising from the source–sink metapopulation structure herein inferred for *C. tricolor* in the Florida Keys. First, our observations imply that future studies aimed at assessing standing stocks or describing other aspects of the life history of this hermit crab need to focus on several localities simultaneously, if an accurate stock estimation and a comprehensive view of the reproductive ecology of this species is to be covered. In many cases, logistics, time, or monetary constraints will not allow such collection-intensive and comprehensive studies. Indeed, other than a few exceptions, natural history studies often focus on a single population (but see Baeza et al., 2013 and references therein for exceptions). When such limitations do not allow exploration of small spatial scale variation in population or reproductive traits, conclusions based on a single-population should be considered carefully as they might not represent the ‘norm’ in small marine invertebrates.

From a management and conservation perspective, our study suggest that any plan to protect this species from over-fishing needs to be carefully designed such that the source–sink dynamic is considered and any protection measures are distributed accordingly. For example, if harvest restrictions were implemented they would be most effective if focused on localities that harbor source populations, in theory, capable of exporting larvae to nearby populations (Buxton et al., 2014). Unfortunately, our lack of knowledge about the temporal dynamics of the studied populations did not allow us to reliably define populations in the Florida Keys acting as sources. Climatic cycles (e.g., El Niño / La Niña) are know to affect reproductive schedules of many marine invertebrates at small, moderate, and large temporal scales (Riascos et al., 2009 and references therein). The effect of oceanographic conditions and cycles on the different populations of *C. tricolor* needs to be understood before any of the populations studied herein can be reliably classified either as a source or sink. Importantly, we also know little about connectivity among the studied populations of *C. tricolor* in the Florida Keys or the type of development (e.g., direct, abbreviated, indirect) exhibited by *C. tricolor*. Other congeneric species are reported to exhibit abbreviated larval development (e.g., *C. aequabilis* and *C. erythropus* passes through five zoeal stages and one megalopa, the latter stage reached after 8 days—Bartilotti, Calado & Santos, 2007 and references therein) but developmental modes and larval periods can differ substantially at low taxonomic levels in many clades of decapod crustaceans (Hernandez et al., 2012). We argue in favor of additional studies describing the temporal dynamics and other aspects of the natural history of *C. tricolor* (e.g., development type, larval duration) in order to improve our understanding of source–sink dynamics in the most heavily traded ornamental crustacean in the Florida Keys.

Lastly, our study also demonstrates considerable small-scale spatial variation in other population parameters of *C. tricolor*, which are of ecological and evolutionary relevance, e.g., sexual dimorphism and sex ratio. With respect to sexual dimorphism, in *C. tricolor*, males attained larger average and final body sizes than females. This pattern of sexual dimorphism (males >females) agrees with that reported for various other congeneric species (e.g., Wait, 2010 and references therein). That males attain larger body sizes than females in *C. tricolor* suggests strong male-male competition for receptive females and the use by males of agonistic interactions during sexual competition as suggested by sexual selection theory (Shuster & Wade, 2003). Studies on the reproductive behavior of both males and females would help reveal the mating system of this ornamental hermit crab.

Sexual dimorphism also varied considerably among *C. tricolor* populations. The differences in body size between male and female hermit crabs were much larger at Money Key and Ohio-Missouri Channel than at Islander Resort, where the differences in body size between the sexes were among the smallest observed across the Florida Keys. At present, we do not have an explanation for such remarkable differences in sexual dimorphism. Nonetheless, given the small body size of *C. tricolor*, and the likely role of predation in structuring populations (Tegner & Levin, 1983; Robles, 1987), differences in size-specific predation rates among sampling sites might explain the observed differences. For instance, more intense predation on large size individuals (mostly males) at the Islander Resort site, but not at the Money Key or Ohio-Missouri Channel sites could explain differences in the extent of sexual dimorphism in this species. Unfortunately, little is known about the vertebrates (including humans) and invertebrates that prey on *C. tricolor*. Additional conditions that might explain our observations include food and/or shell size availability that differ among the different study sites. Field observations on food resources, shell availability, and predators of this hermit crab coupled with experiment to determine the effect of body size, sex, and their interaction on crab mortality are needed to understand differences in sexual dimorphism not only in this species but in other small marine invertebrates.

Finally, in *C. tricolor*, sex ratio also varied considerably among the studied populations. Among four populations, the sex ratio did not differ significantly from a 1:1 sex ratio, in two others the sex ratio was biased towards males and in three others it was biased toward females. In species with separate sexes, such as *C. tricolor*, sex allocation theory predicts that parents should produce an equal number of male and female offspring (primary sex ratio) because of frequency-dependent selection against the more common sex in the population (Fisher, 1930; Charnov, 1982; West, 2009). Our results disagree with predictions at the core of evolutionary theory, but admittedly, we did not measure the primary sex ratio but the total population sex ratio. The conditions explaining deviations from a 1:1 sex ratio in more than half of the studied populations of *C. tricolor* were enigmatic and remain to be addressed.

## Conclusion

We have shown remarkable dissimilarity in terms of abundance and reproductive traits at small spatial scales in the hermit crab *C. tricolor*, a small marine invertebrate heavily exploited by the ornamental industry. The observed disparity in abundance, prevalence of reproductive females, fecundity, and sex ratio among the studied populations suggests that *C. tricolor* exhibits a source–sink metapopulation dynamic in the Florida Keys. These results exemplify the importance of gaining a thorough understanding of the life history, population dynamics, and reproductive biology of a species under heavy exploitation. We suggest that additional studies on the temporal dynamics of the system and connectivity among populations are needed to reliably identify source and sink populations, but perhaps most importantly, our study sites in the Florida Keys were not located in areas protected from harvest. There is no evidence that ornamental fishermen target *C. tricolor* at a particular site, but regardless, future studies should consider measuring population and reproductive parameters in protected and unprotected areas along the Florida Keys. Such studies would reveal whether or not some of these protected areas are already providing conservation benefits and further support informed sustainable management.

##  Supplemental Information

10.7717/peerj.3004/supp-1 Data S1

## Additional Information and Declarations

### Field Study Permissions

The following information was supplied relating to field study approvals (i.e., approving body and any reference numbers): Field collections were approved by FWCC (permit number: SAL-11-1319-SR).

### Data Availability

The following information was supplied regarding data availability: The raw data is located in Figs. 2–4 and has been supplied as a Data S1.
